# Drinking Warm Water Improves Growth Performance and Optimizes the Gut Microbiota in Early Postweaning Rabbits during Winter

**DOI:** 10.3390/ani9060346

**Published:** 2019-06-12

**Authors:** Qiangjun Wang, Wei Fu, Yao Guo, Yuhan Tang, Haoxuan Du, Meizhi Wang, Zhongying Liu, Qin Li, Lei An, Jianhui Tian, Mingyong Li, Zhonghong Wu

**Affiliations:** 1 State Key Laboratory of Animal Nutrition, College of Animal Science and Technology, China Agricultural University, Beijing 100193, China; wangqiangjun182@163.com (Q.W.); fuwei11223344@163.com (W.F.); guoyaocau@163.com (Y.G.); xmztyh999@163.com (Y.T.); meizhiwang@cau.edu.cn (M.W.); LZY228@cau.edu.cn (Z.L.); crystal_stefer@126.com (Q.L.); 2 ZhaoTong Technology Promotion Workstation of Animal Husbandry and Veterinary Medicine, ZhaoTong 657000, China; 3 National Engineering Laboratory for Animal Breeding, College of Animal Science and Technology, China Agricultural University, Beijing 100193, China; haoxuandu188@163.com (H.D.); anleim@cau.edu.cn (L.A.); tianjh@cau.edu.cn (J.T.); 4 National Rabbit Industry Technology System Qingdao Comprehensive Experimental Station, Qingdao 266431, China; lmy77@126.com

**Keywords:** cecal microbiota, rabbit, water temperature, gut health, welfare, winter

## Abstract

**Simple Summary:**

Epidemiological investigations have revealed that cold temperature can increase the risk of diarrhea in children, and similar observations have been made in early postweaning livestock. Recent studies have shown that cold temperature alters the gut microbiota, which may be associated with the pathogenesis of various intestinal diseases, such as inflammatory bowel diseases. However, few studies have focused on how to improve intestinal health from the perspective of the gut microbiota in early postweaning livestock during winter. In the present study, using early postweaning rabbits as a model, we analyzed the effects of drinking warm water (WW) on the growth performance and gut microbiota structure of postweaning rabbits during winter. Our results confirmed that drinking WW improved the growth performance and optimized gut microbiota in early postweaning rabbits during winter. Therefore, our results provide a new perspective for improving early postweaning rabbits welfare during winter.

**Abstract:**

Accumulating evidence indicates that cold exposure changes the composition of the gut microbiota and reduces intestinal immunity in early postweaning livestock. However, little is known about the effects of drinking warm water (WW) on gut microbiota during winter. In this study, we investigated the effects of drinking WW in winter on the growth performance and gut microbiota structure of rabbits raised in poorly insulated housing from the early postweaning period (day 46) to the subadult period (day 82). The average daily gain and feed conversion ratio in rabbits drinking WW were significantly improved compared to those of the rabbits drinking cold water (CW) during 47–58 days. In addition, rabbits drinking WW had a significantly decreased the risk of diarrhea during 71–82 days. 16S rRNA sequence analysis revealed that the alpha diversity of the cecal microbiota was not significantly different between the WW and CW groups, but significantly increased with age. The relative abundance of cecal microorganisms, such as *Coprococcus* spp. was considerably increased at day 70 in the group drinking WW. Correlation analysis indicated that *Coprococcus* spp. was negatively associated with pro-inflammatory factors. In conclusion, our results suggest that drinking WW has a positive effect on growth performance and gut microbiota in rabbits during the early postweaning stage in winter.

## 1. Introduction

The balance of gut microbial composition is critical for host physiology, and disruption to its composition has been associated with metabolic disorders and gastrointestinal diseases, such as obesity and inflammatory bowel diseases (IBD), encompassing ulcerative colitis (UC) and Crohn’s disease (CD) [1,2]. Recent studies have indicated that cold temperature can change the composition and function of the gut microbiota [3,4]. In addition, cold temperature can also impede human health, especially in children who experience a greater risk of gastrointestinal diseases, such as diarrhea, under colder conditions [5,6]. Similar effects have been reported in early postweaning livestock, who suffer from both cold stress and weaning stress during winter, leading to higher mortality and risk of diarrhea, lower growth rates, and increased economic losses in farmed animals [7,8]. Previous studies have revealed that low-temperature environments and weaning stress impaired intestinal mucosal immunity, barrier function, and the homeostasis of cecal microflora [9,10,11]. One of the major contributors to these effects is that the deprivation of the mother’s milk after weaning causes weaned animals to lose the passive gut immunity provided by antibodies in breast milk [12]. At the same time, the weaned animals switch from warm breast milk to cold water, and their intestinal health is affected by stress from both the diet change and low temperature [13]. A previous study indicated that optimal gut microbiota can maintain intestinal health and then promote the production performance of animals [14]. Interestingly, drinking warm water or warm milk replacers has been found to have a beneficial effect on the growth performance of animals during winter [15,16]. However, it is not clear whether drinking WW promotes gut health by altering gut microbes.

The mammalian gut microbiota plays vital roles in maintaining intestinal homeostasis, resisting the invasion of pathogenic microorganisms, and stimulating the development of the immune system [17,18]. From birth to adulthood, the gut microbiome of animals develops from the relatively simple maternally acquired and unstable community to more complex communities due to diet switching from breast milk to solid food and dietary supplements [19]. This gradual shift in microbial communities is consistent with the rapid development of the immune system [20]. In addition, along with the development of the microbiota, microbial communities produce more fermentation products, such as short-chain fatty acids (SCFAs), mainly acetate, propionate, and butyrate, which play important roles in modulating inflammation, healing wounds, and gut motility [21]. Among the SCFAs, butyrate not only acts as an energy source for intestinal epithelial cells, but also serves as a key mediator of anti-inflammation and intestinal homeostasis [22,23]. Previous studies have revealed that butyrate suppresses intestinal inflammation through GPR109a-mediated differentiation of T regulatory (T_Reg_) cells [22,23,24]. Furthermore, SCFAs could improve the expression of intestinal tight junction genes, which were effective in enhancing the integrity of the intestinal barrier and warding off pathogen invasion [25,26].

Recently, many studies have been conducted to evaluate the effect of low temperature on the gut microbiota. In mice, cold exposure could lead to significant changes in the composition of the gut microbiota, and both host insulin sensitivity and tolerance to cold were enhanced when the gut microbiota from cold-exposed mice was transplanted into germ-free mice [3]. Similarly, Zhang et al. [27] found that the coevolutionary mechanism between the remodeled gut microbiota and the host was associated with thermoregulation and energy-saving during winter. Numerous studies on livestock have revealed that probiotics can be efficacious in modulating the effects of cold stress on gut health in monogastric animals [28,29]. However, although previous studies have suggested that cold stress can affect health by remodeling the composition of the gut microbiota, the influence of drinking WW on alleviating these effects of cold stress and weaning stress by remodeling gut microbes has not been well documented.

In the present study, we investigated the effects of drinking WW in winter on the gut health of early postweaning rabbits from the perspective of intestinal immunity, cecum fermentation, and gut microbiota. Rabbits have been proposed as a model animal to study a number of human diseases, including intestinal diseases, owing to their anatomical and physiological similarity to primates, more so than other rodents, such as mice and rats [30]. Other advantages include their small size and low feeding cost to produce lower-fat and higher-protein meat compared to other livestock [31]. Our findings provide evidence to support the drinking of WW by newborn and weaned animals in winter to improve gut health.

## 2. Materials and Methods

### 2.1. Ethics Statement

The rabbits used in this study were maintained according to the guidelines of the China Agricultural University Animal Care and Use Ethics Committee (AW15049102-1). All management and experimental procedures were carried out in accordance with the relevant guidelines and regulations.

### 2.2. Animal Management and Sample Collection

Rabbits were raised in a poorly insulated barn (open barn enclosed with plastic curtain) at the experimental station of the National Technology System of Rabbit Industry (Qingdao, China) during winter (December 2015–January 2016). The average indoor and outdoor temperature was 7.8 ± 0.9 °C and 0.3 ± 3.7 °C, respectively, throughout the experimental period. A total of 180 Ira early postweaning rabbits (46 days of age) with similar body weight (initial body weight of 1.2 ± 0.1 kg) were randomly assigned to groups that were given either warm water (WW) or cold water (CW). The WW was heated by an electrical heating wire (SN-EA, Yangzhou, China) wrapped around the water pipe covered with insulated foam coating, and was controlled at a constant temperature (35.5 ± 1 °C) with a thermostatic controller (HY-WKX-3, Taizhou, China) during the course of the experiments. The CW was 5.8 ± 2.3 °C without heating. Rabbits were caged in individual units (cage size: 60 cm × 62 cm × 30 cm) and the individual feed intake was recorded daily. Rabbits could drink water and eat standard pellets *ad libitum*. The diet was formulated to contain the predicted nutrient requirements lacking antibiotics and medicines. The composition and nutrient content of the commercial pellet feed is presented in Table S1.

In this study, we were focused on the effects of WW and CW during the rabbit growth stage. Therefore, the rabbits were weighed every 12 days from 58 days of age (58, 70, and 82 days of age) [19,32]. The feed conversion ratio was calculated as the ratio of feed intake/body weight gain during different stages [33]. Meanwhile, 15 healthy rabbits were randomly selected from WW or CW group and euthanized by cervical dislocation. The cecum was quickly dissected, the pH of the cecum content was measured with a pH meter (S8-Meter, Shanghai, China), and the fresh cecal contents were gently squeezed and carefully collected in 1 mL sterile tubes, frozen in liquid nitrogen, and then stored at −80 °C for further analysis. The middle segments of the jejunum were collected for examining intestinal morphology and detecting gene expression.

### 2.3. Intestinal Histological Analysis

Samples of jejunum segments from each rabbit were immediately fixed in 4% (v/v) polyformaldehyde, and then embedded in paraffin, and 5-µm sections were stained with hematoxylin and eosin [34]. Images of the morphology of the jejunum were collected using Visitron Systems (GmbH, Puchheim, Germany). Villus height and crypt depth were measured from these images and analyzed using NIS-Elements Basic Research software, version 2.20 (Nikon, Tokyo, Japan).

### 2.4. SCFAs and NH_3_-N Determination

The concentrations of SCFAs were determined by gas chromatography as previously described [35]. Briefly, approximately 1 g of thawed cecal contents were diluted in a screw-capped tube with 1.5 mL of sterile distilled water and then centrifuged at 10,000× *g* for 10 min at 4 °C. One milliliter of the supernatant was mixed with 300 μL of 25% (w/v) metaphosphoric acid solution in an ampoule. After periodic vortexing, the ampoules were incubated at 4 °C for 30 min. After being centrifuged at 10,000× *g* again for 10 min, the supernatant sample was injected into a HP 6890 Series Gas Chromatograph (Hewlett Packard, Palo Alto, CA, USA), which was equipped with an HP 19091N-213 column with a 30 m × 0.32 mm id (Agilent, Santa Clara, CA, USA). The injector temperature was 185 °C and the detector temperature was set to 210 °C. The injected sample volume was 0.5 μL. The concentration of NH_3_-N was determined by phenol-hypochlorite colorimetry [36]. Briefly, 1 g of thawed cecal contents were diluted in 1.5 mL of sterile distilled water, and then centrifuged at 10,000× *g* for 10 min at 4 °C. One milliliter of the supernatant was mixed with 150 μL of 65% (w/v) metaphosphoric acid solution and quantified at 635 nm.

### 2.5. Extraction of RNA and Real-Time Quantitative PCR Analysis (qPCR)

Total RNA was extracted from the jejunum using the TRIzol reagent (Invitrogen, Carlsbad, CA, USA), following the manufacturer’s protocols. mRNA expression was measured in a CFX96 real-time PCR system (Bio-Rad, Hercules, CA, USA) using SsoFast EvaGreen Supermix. Three replicates were performed, and the relative mRNA expression of target genes was assessed using the comparative cycle threshold method and normalized to GAPDH expression. Primer pairs are listed in Table S2.

### 2.6. Cecal Contents DNA Extraction and Sequencing

Total bacterial DNA was extracted from each cecal content sample using the Power Soil DNA Isolation Kit (MO BIO Laboratories, Carlsbad, CA, USA) according to the manufacturer’s instructions. DNA was stored at −80 °C until further processing. The bacterial universal V3–V4 region of the 16S rRNA gene was amplified with the common primer pair (forward primer, 5′-ACTCCTACGGGAGGCAGCA-3′; reverse primer, 5′-GGACTACHVGGGTWTCTAAT-3′). After amplification, high-throughput sequencing of bacterial rRNA genes was performed using the Illumina HiSeq 2500 platform (Biomarker, Beijing, China). The sequencing data were uploaded into the Sequence Read Archive (SRA) of NCBI and can be accessed from NCBI SRA (BioProject ID: PRJNA512067).

### 2.7. Bioinformatics Analysis

Clean reads were assembled, and chimeric reads were discarded using UCHIME (v. 4.2) [37]. Sequences were binned into operational taxonomic units (OTUs) based on 97% identity using UCLUST (v. 1.2.22) [38] after removing the singletons, and then the OTUs with sequences less than five in 100,000 of total sequences were filtered out according to a previous study [39]. In addition, to eliminate the effects of sequence number variation from different samples, we rarefied each sample to the minimum sequencing depth through a subset of randomly selected reads prior to downstream analysis; this rarefaction was performed using a single_rarefaction.py script in QIIME [40]. Based on Silva [41], the Ribosomal Database Project classifier was applied to the taxonomic analysis. Mothur software (v. 1.30.1) [42] was applied to the diversity indices analysis, which included community richness (Chao1 and Ace) and diversity (Shannon and Simpson). Linear discriminant analysis (LDA) coupled with effect size measurement (LEfSe) analysis was used according to the online protocol (https://huttenhower.sph.harvard.edu/galaxy/). Correlation network analysis between the gut immune-related indices and microbiota was performed using Spearman’s rho non-parametric statistic, and the results were graphically visualized using Cytoscape (v. 3.5.1) [43]. The data for Spearman’s rho non-parametric test were obtained from the WW groups (including 58, 70 and 82 days of age) and CW groups (including 58, 70 and 82 days of age), respectively.

### 2.8. Statistical Analysis

Data analysis was performed using SPSS 20.0 (SPSS, Inc., Chicago, IL, USA). The effects of age and water temperature on alpha diversity and fermentation parameters were analyzed by two-way analysis of variance (ANOVA). Bacterial compositional nonparametric data were used for a Wilcoxon rank-sum test. The odds ratio (OR) was used to evaluate the incidence of diarrhea, and the chi-square test was calculated with a 95% confidence interval (95% CI). For all other data, an unpaired t-test was used to analyze differences between the two groups. One-way ANOVA with the least-significant difference *post hoc* test was used for multiple comparisons. Differences at *p* < 0.05 were considered statistically significant. In addition, all *p* values from the multiple comparison analyses of the bacterial community (genus level) and gut immune-related indices were adjusted by the FDR with *p* adjust; FDR-corrected *p* values < 0.05 (*q* < 0.05) were considered to indicate a statistically significant difference. Figures were generated in Prism 7.0 (GraphPad Software Inc., La Jolla, CA, USA).

## 3. Results

### 3.1. Growth Performance and Gut Morphology Changes within Warm- and Cold-Water Groups

**Figure 1:**
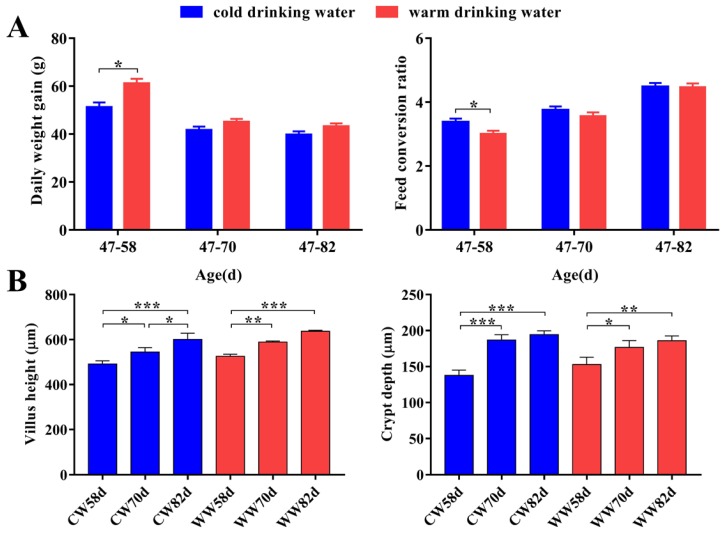
Effects of drinking warm water on the production performance and intestinal mucosal morphology of rabbits. CW, cold water group; WW, warm water group. (**A**) Average daily weight gain and feed conversion ratio (*n* = 15). (**B**) The villus height and crypt depth in the jejunum (*n* = 4–8). * *p* < 0.05, ** *p* < 0.01, *** *p* < 0.001. Values are shown as means ± SEM. Differences were analyzed by one-way analysis of variance followed by the least-significant difference *post hoc* test.

The average daily weight gain and feed conversion ratio (FCR) of rabbits in the WW group were significantly higher than those of rabbits in the CW group during 47–58 days (Figure 1A), but were not significantly different during 47–70 and 47–82 days. Compared with the CW group, rabbits drinking WW had a significantly decreased the risk of diarrhea during 71–82 days (Table 1). In addition, the villus height and crypt depth of the intestinal mucosa increased with age (Figure 1B), but no significant difference was observed between the WW and CW groups at the same age stage.

### 3.2. Expression of Intestinal Immunity and Tight Junction-Related Genes in Warm- and Cold-Water Groups

**Figure 2:**
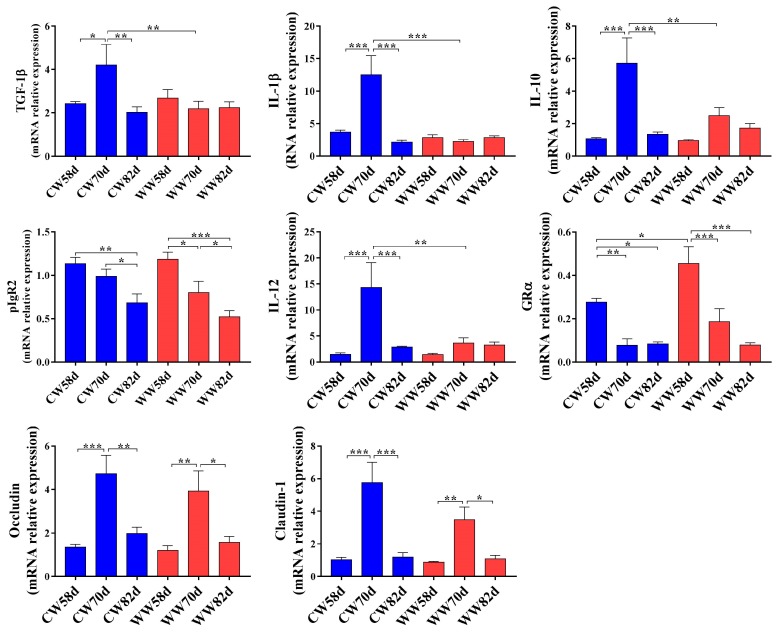
Effects of drinking warm water on intestinal immune function and expression of tight junction genes (*n* = 8). CW, cold water group; WW, warm water group. * *p* < 0.05, ** *p* < 0.01, *** *p* < 0.001. Values are shown as means ± SEM. Differences were analyzed by one-way analysis of variance followed by the least-significant difference *post hoc* test.

The effects of drinking WW on cytokine and tight junction-associated gene expression in the jejunum are shown in Figure 2. The expression of *TGF-1β*, *IL-1β*, *IL-10*, and *IL-12* was significantly reduced in the WW group compared to those of the CW group at day 70. However, the expression of these genes was not significantly different between the WW and CW groups at days 58 and 82. Similarly, we analyzed the expression of *Occludin* and *Claudin-1* related to the intestinal barrier in the jejunum with no significant differences observed between the WW and CW groups at days 58, 70, and 82. Furthermore, glucocorticoids exhibit anti-inflammatory activity through glucocorticoid receptors, and the expression of glucocorticoid hormone receptor alpha (*GRα*) in jejunum tissues from WW rabbits was significantly higher than that of CW rabbits at day 58. In addition, *pIgR2* is associated with innate and adaptive immunity, which increases in response to bacterial or viral infections. With increasing age, the expression of *pIgR2* and *GRα* in the jejunum were significantly decreased in both groups.

### 3.3. Fermentation Changes within Warm- and Cold-Water Groups

**Figure 3:**
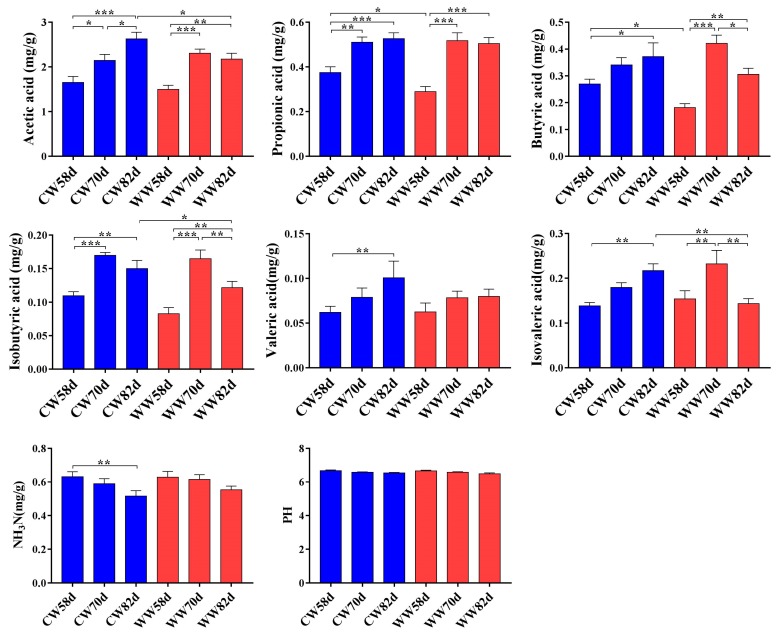
Effects of drinking warm water on short-chain fatty acids (SCFAs) concentration, NH_3_-N concentration, and pH values of rabbits (*n* = 8). CW, cold water group; WW, warm water group. * *p* < 0.05, ** *p* < 0.01, *** *p* < 0.001. Values are shown as means ± SEM. Differences were analyzed by one-way analysis of variance followed by the least-significant difference *post hoc* test.

To explore the effect of the temperature of drinking water and age on cecal fermentation, SCFAs, NH_3_-N, and pH were analyzed by two-way ANOVA (Table 2). The concentrations of SCFAs and NH_3_-N were mainly affected by age, except for the concentration of isobutyric acid, which was also influenced by water temperature. Interestingly, we found a significant interaction effect of water temperature and age on the concentrations of butyric acid and isovaleric acid. Furthermore, the concentrations of SCFAs, such as acetic acid, propionic acid, and butyric acid, increased with age (Figure 3). The concentrations of propionic acid and butyric acid were markedly increased at day 58 in the CW group compared with those of WW group, but there was no significant difference between the two groups at other age stages.

### 3.4. Microbial Diversity Changes within Warm- and Cold-Water Groups

**Figure 4:**
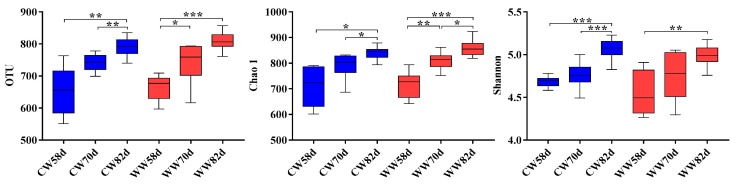
Effects of drinking WW on the diversity of the cecal bacterial community (*n* = 9–15). CW, cold water group; WW, warm water group. Values are shown as means ± SEM. Differences were analyzed by one-way analysis of variance followed by the least-significant difference *post hoc* test. * *p* < 0.05, ** *p* < 0.01, *** *p* < 0.001.

To further study the effect of drinking WW on gut microbial diversity at different age stages, alpha diversity, including OTU, ACE, Chao1, Simpson, and Shannon indices, were analyzed by two-way ANOVA (Table 3). We found that alpha diversity was mainly affected by age, especially with regards to indices of species richness (Chao1) and diversity (Shannon), both of which significantly increased with host’s age (Figure 4).

### 3.5. Microbial Community Composition Changes within Warm- and Cold-Water Groups

**Figure 5:**
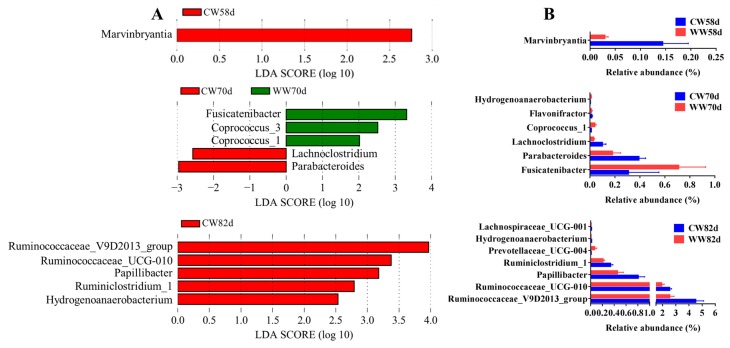
Alteration of the microbial genera among the different groups (*n* = 9–15). (**A**) Genera that are differentially represented between the groups drinking warm and cold water identified by LEfSe (linear discriminant analysis score > 2, *p* < 0.05). (**B**) Significantly different genera among the different groups are shown, based on *p* < 0.05 by the Wilcoxon rank-sum test. CW, cold water group; WW, warm water group.

The dominant microbial phylum in the cecal microbiota was Firmicutes followed by Bacteroidetes, Verrucomicrobia, and Proteobacteria. At the genus level, Firmicutes mostly comprised *Lachnospiraceae_NK4A136_group*, *Ruminococcaceae_V9D2013_group*, and *Ruminococcaceae_NK4A214_group* (Figure S1). LEfSe and Wilcoxon rank sum test analyses were applied to detect significantly different microorganisms between the two groups (Figure 5). At day 58, the relative abundance of cecal *Marvinbryantia* from rabbits drinking WW was significantly reduced compared to that of rabbits drinking CW. At day 70, the relative abundance of microorganisms, such as *Fusicatenibacter*, *Coprococcus_1*, and *Coprococcus_3*, were significantly higher in the cecum of rabbits drinking WW than that of rabbits drinking CW. In contrast, the relative abundance of microorganisms, such as *Lachnoclostridium* and *Parabacteroides*, were significantly reduced in the cecum of rabbits drinking WW. At day 82, we found that WW rabbits had a distinctively lower abundance of *Ruminococcaceae_V9D2013_group* and *Ruminococcaceae_UCG-010* than that of the CW rabbits (Figure 5). Furthermore, we found that the relative abundance of beneficial microorganisms, such as *Ruminococcaceae_UCG-009* and *Coprococcus* spp., markedly increased with age (Figure S2).

### 3.6. Correlations between the Gut Microbiota and Immunity-Related Genes

**Figure 6:**
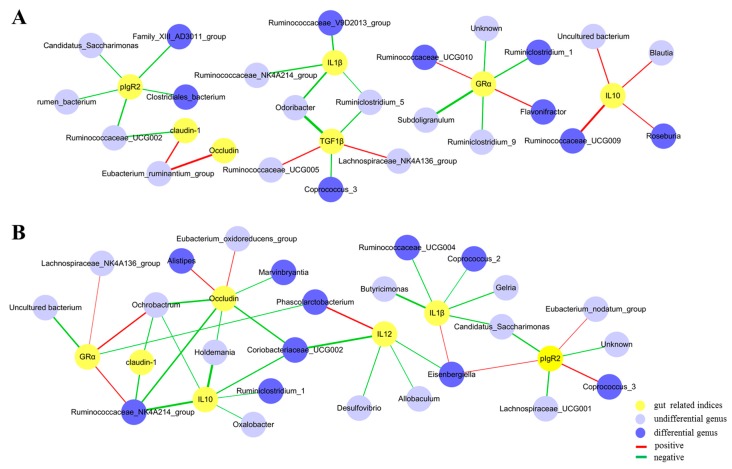
Correlation analysis between microbiota abundance and gut-related indices. Spearman’s rho non-parametric correlation was applied, and significant relationships with *q* < 0.05 are shown. (**A**) Correlation between the microbiota and gut-related indices in WW groups. Yellow nodes: Gut-related indices, referring to immune and tight junction-associated gene expression in WW groups. Blue nodes: Differential genus, referring to the genera that significantly differed between the groups (WW vs. CW) or markedly increased with age (day 58 vs. 82) in WW groups. Light blue nodes: Undifferential genus, referring to genera that were not significantly different between the groups (WW vs. CW) or did not markedly increase in abundance with age (day 58 vs. 82) in WW groups. (**B**) Correlation between the microbiota and gut-related indices in CW groups. Yellow nodes: Gut related indices, referring to immune and tight junction-associated gene expression in CW groups. Blue nodes: Differential genus, referring to the genera that significantly differed between the groups (WW vs. CW) or markedly increased with age (day 58 vs. 82) in CW groups. Light blue nodes: Undifferential genus, referring to the genera that were not significantly different between the groups (WW vs. CW) or did not markedly increase in abundance with age (day 58 vs. 82) in CW groups. Red lines indicate positive correlations between nodes (r ≥ 0.4), and green lines indicate negative correlations (r ≤ −0.4). The thickness of the lines is proportional to the obtained r values. CW, cold water group; WW, warm water group.

To further investigate the effect of drinking WW on microbial community composition of the cecum, we analyzed the correlation between gut-related indices and the relative abundance of microorganisms at the genus level. *TGF1β*, as a pro-inflammatory cytokine, was negatively associated with *Coprococcus_3* (r = −0.48, *q* < 0.05) in WW groups (Figure 6A). *IL-1β*, as a pro-inflammatory cytokine, was negatively associated with *Ruminococcaceae_V9D2013_group* (r = −0.52, *q* < 0.05) in WW groups. In addition, *IL-10*, as an anti-inflammatory cytokine, was positively associated with *Ruminococcaceae_UCG-009* (r = 0.63, *q* < 0.05) in WW groups. In contrast, *IL-1β* was negatively related to *Coprococcus_2* (r = −0.40, *q* < 0.05) and *Ruminococcaceae_UCG-004* (r = −0.44, *q* < 0.05) in CW groups (Figure 6B). Moreover, *IL-10* was negatively related to *Ruminococcaceae_NK4A214_group* (r = −0.61, *q* < 0.05) and *Ruminiclostridium-1* (r = −0.41, *q* < 0.05) in CW groups (Figure 6B).

## 4. Discussion

Evidence has indicated that cold stress and weaning stress shift the gut microbiota, which can lead to poor growth performance and gut health by altering the composition of gut microbiota [3,4,44]. However, few studies have focused on how to improve intestinal health from the perspective of the gut microbiota in early postweaning livestock during winter. In the present study, we analyzed the effects of drinking WW on rabbit growth performance, gut immune function, cecum fermentation, and microbiota structure during winter. We found that the average daily gain and FCR in early postweaning rabbits (day 58) were considerably improved by drinking WW. The jejunal mRNA expression of pro-inflammatory cytokines (TGF1β, *IL-1β* and *IL-12*) was significantly attenuated in the WW group compared to that of the CW group at day 70. High-throughput 16S rRNA sequencing revealed that some of the beneficial microorganisms, such as *Coprococcus_1* and *Coprococcus_3*, increased in WW rabbits at day 70. Therefore, this study revealed that the beneficial effects of drinking WW partially resulted from altering the composition of gut microbiota.

Early colonizing intestinal microbes are affected by a variety of factors, such as whether the animal is fed breast milk or formula, antibiotics, and solid food after weaning [45]. In this study, the cecum microbial composition of weaned rabbits also changed by drinking WW in winter. For example, the relative abundances of *Coprococcus_1*, *Coprococcus_3*, and *Fusicatenibacter* in the cecum of rabbits drinking WW at day 70 were significantly higher than that of rabbits drinking CW. The decreasing abundance of *Coprococcus* spp., an anaerobic microbe, has been shown to negatively affect host health [46]. Previous studies have also found that pro-inflammatory factors can reduce the relative abundance of *Coprococcus* spp., and that its low abundance is associated with the occurrence of IBD [47]. In this study, correlation network analysis found that *Coprococcus* spp. was significantly negatively correlated with the pro-inflammatory factor *IL-1β* and *TGF1β*, respectively. Similarly, the relative abundance of *Fusicatenibacter* is significantly reduced in the feces of UC patients, and *Fusicatenibacter* fed to mice could alleviate colon inflammation [48]. These results suggested that drinking WW might promote host intestinal health by increasing the relative abundance of *Coprococcus_1*, *Coprococcus_3*, and *Fusicatenibacter*. In contrast, the relative abundances of *Lachnoclostridium* and *Parabacteroides* were significantly higher in the cecum of rabbits drinking CW than rabbits drinking WW at day 70. Qiu et al. (2017) found that the relative abundance of *Lachnoclostridium* significantly increased in samples of UC patients [49]. The correlation network analysis revealed a significant positive correlation between *Lachnoclostridium* and *IL-1β* [50]. In addition, *Parabacteroides* is associated with *Clostridium* difficile-positive diarrhea and CD [51,52]. A previous study found that the intestinal microorganisms of young animals were still in the “window stage” during the early weaned stage, and that changes in intestinal microorganisms during this period will increase the susceptibility to diseases at later ages [53]. Consistent with these results, we found that the risk of diarrhea was lower in rabbits drinking WW at days 71–82. Therefore, these results suggested that WW could optimize the intestinal microflora of rabbits by increasing the relative abundance of beneficial microorganisms and reducing the relative abundance of pathogenic microorganisms in winter, thereby reducing the risk of diarrhea of subadult rabbits. However, these findings require further experimental confirmation.

Intestinal health is related to the richness and diversity of intestinal microorganisms [54]. Here, we study found that the alpha diversity of rabbit cecum microorganisms significantly increased with rabbit age, which was consistent with previous studies on rabbits and pigs [19,55]. Previous studies have found that a high diversity of intestinal microbes has been regarded as a sign of intestinal microbial maturity [56], and it is generally considered to be beneficial for host health. This is considered to be related to the fact that a high level of intestinal microbial diversity can help the intestinal ecosystem maintain its resilience, resistance, and stability when the host suffers environmental pressure [57]. Furthermore, this study found that the relative abundance of *Ruminococcaceae_UCG-009* and *Coprococcus* spp. increased with the age of rabbits, which were related to promoting intestinal health. *Ruminococcaceae_UCG-009* and *Coprococcus* spp. produce butyric acid by degrading fructose, and *Coprococcus* spp. also produce propionic acid via the acrylate pathway [58]. Previous studies indicated that the anti-inflammatory activity of propionic acid and butyric acid can be activated by inhibiting the histone deacetylases of macrophages and dendritic cells. Moreover, butyric acid also plays an anti-inflammatory role in promoting the differentiation of T_Reg_ cells and CD4 + CD25 + T cells [59,60]. Our network analysis found that *Ruminococcaceae_UCG-009* had a significant positive correlation with the anti-inflammatory factor *IL-10*. These results suggested that cecal microorganisms become more mature with increasing age of the rabbit, and then intestinal homeostasis could be maintained by increasing the relative abundance of microorganisms associated with anti-inflammatory responses. This could avoid the influence of drinking CW on intestinal microflora in winter.

Low-temperature stress affects the production performance and health of rabbits [61]. This experiment found that the average daily gain and FCR of rabbits drinking CW were significantly lower than those of rabbits drinking WW at day 58. Another study found that the growth performance of animals is affected by the integrity of the intestinal barrier function, and also by the length of the villi and depth of the crypts [14]. However, in the present study, there was no significant difference in the expression of jejunal tight junction genes, *Occludin* and *Claudin-1*, as well as the villus length and crypt depth between the WW and CW groups when the rabbits were 58 days old. These results suggested that the better production performance of early weaned rabbits promoted by drinking WW was concerned with reducing the energy loss of the digestive tract, which was used to resist the cold stress [62,63]. Interestingly, the relative abundance of *Marvinbryantia* in the cecum of rabbits in the CW group was significantly higher than that in the WW group at day 58. This microorganism belongs to *Lachnospiraceae*, which can produce propionic acid and butyric acid by degrading non-starch polysaccharides through the acrylate pathway and butyric acid kinase pathway, respectively [58,64]. This is consistent with the result that the concentrations of propionate and butyrate in the cecum of rabbits in the CW group were significantly higher than those in the WW group at this age stage. Propionic acid and butyric acid are important energy metabolites, which provide energy for animal growth [65]. As a substrate for gluconeogenesis, propionic acid can be absorbed by monocarboxylate transporters on intestinal epithelial cells and then transported to the liver for glucose synthesis. Butyric acid is absorbed by intestinal epithelial cells and then directly provides energy through mitochondrial oxidation to maintain the energy balance of intestinal epithelial cells [66]. These findings suggest that higher concentrations of propionic and butyric acids might be used as an energy source to resist heat loss in the digestive tract when the rabbit drinks CW in winter [62,63]. These findings were similar to recent studies in mice, which found that cold exposure can be resisted by increasing the concentration of acetic acid and propionic acid in the cecum [3]. In addition, glucocorticoid plays an important role in regulating intestinal metabolism and immunity [67,68]. The present study showed that the mRNA expression of *GRα* in the jejunum was significantly higher in the WW group than in the CW group on day 58. Glucocorticoids combined with glucocorticoid receptors can increase the metabolism of glutamine in intestinal epithelial cells of weaned piglets [67], and glutamine is used as an energy substrate to provide energy for the rapid proliferation of intestinal epithelial cells and T lymphocytes [69]. Furthermore, glucocorticoids also suppress the NF-κB signaling pathway through their receptors to reduce the expression of pro-inflammatory cytokines in the gut [68]. Taken together, these results indicated that rabbits drinking WW in winter could not only reduce the energy loss from the digestive tract, but could also increase the metabolism of nutrients and the immune response in the gut through GR, and subsequently promote the healthy growth of weaned young rabbits.

Intestinal mucosal immune balance is essential for livestock health [14]. Compared with the CW group, this study found that the expression of *IL-1β* and *IL-12* mRNA in the jejunum were significantly reduced in the WW group at day 70. Previous studies have found that drinking CW can lower the temperature of the digestive tract at low temperatures [62,63]. As a temperature sensor, transient receptor potential (TRP) channels induce the release of intracellular Ca^2+^ to activate TAK1, which in turn activates the NFκB signaling pathway and then promotes the expression of inflammatory factors [70]. In addition, microbial products, such as lipopolysaccharide, can activate the Toll-like receptor signaling pathway, which also activates the downstream TAK1/NFκB signaling pathway through the adaptor molecule MyD88 to induce the expression of inflammatory cytokines [71]. Therefore, it is possible that drinking WW could intervene with the NFκB signaling pathway by altering the microbial floral structure and TRP channel activity, and then reduce the expression of intestinal inflammatory factors in weaned rabbits. However, the details of this molecular mechanism require further investigation.

## 5. Conclusions

This study revealed that the growth performance of rabbits in the WW group was significantly higher than that of rabbits in the CW group during 47–58 days. In addition, rabbits drinking WW had significantly decreased the risk of diarrhea during 71–82 days. Furthermore, the relative abundance of cecal microorganisms, such as *Coprococcus* spp., was considerably increased during the early postweaning stage in the group that drank WW, and suppressed the relative abundance of microorganisms, such as *Lachnoclostridium*. Therefore, these findings provide a new perspective on how WW improves the growth performance and optimize gut microbiota in early postweaning rabbits during winter. Our results suggest that drinking WW could be an effective strategy to improve young livestock welfare in winter.

## Figures and Tables

**Table 1:** Odds ratios for the incidence of warm water-associated diarrhea.

Age (d)	Number of Rabbits with Diarrhea/Total Number of Rabbits		Odds Ratio	95% CI ^3^	*p* ^4^
	WW ^1^	CW ^2^			
Day 47 to 58	16/90	20/90	0.76	0.36–1.58	0.46
Day 59 to 70	29/74	31/75	0.92	0.48–1.76	0.79
Day 71 to 82	2/59	14/60	0.12	0.03–0.53	<0.01

^1^ WW, warm water group. ^2^ CW, cold water group. ^3^ 95% CI, 95% confidence interval. ^4^ Chi-square test.

**Table 2:** Significant effects of age and water temperature on small chain fatty acid concentration, NH_3_-N concentration, and pH values.

Item	Main Effects Two-Way ANOVA		
	Age	Water Temperature	Interaction
Acetic acid	***	NS	NS
Propionic acid	***	NS	NS
Butyric acid	***	NS	*
Isobutyric acid	***	**	NS
Valeric acid	*	NS	NS
Isovaleric acid	*	NS	**
NH_3_-N	**	NS	NS
pH	NS	NS	NS

*** *p* < 0.001; ** *p* < 0.01; * *p* < 0.05; NS, *p* > 0.05.

**Table 3:** Age-related change of richness and diversity of the cecal microbiota.

Alpha Diversity	Main Effects Two-Way ANOVA		
	Age	Water Temperature	Interaction
OTUs	***	NS	NS
ACE	***	NS	NS
Chao	***	NS	NS
Simpson	**	NS	NS
Shannon	***	NS	NS

*** *p* < 0.001; ** *p* < 0.01; * *p* < 0.05; NS, *p* > 0.05. OTU, operational taxonomic unit.

## Supplementary Materials

The following are available online at https://www.mdpi.com/2076-2615/9/6/346/s1, Figure S1: Taxonomic alteration of cecum microbiota in rabbits from different groups at the phyla and genus level, Figure S2: Genus differentially represented between the drinking warm and cold water group identified by LEfSe (linear discriminant analysis score > 2, *p* < 0.05), Table S1: Composition of the basal diets, Table S2: Primers used for PCR.

## References

[1] Ridaura V.K., Faith J.J., Rey F.E., Cheng J., Duncan A.E., Kau A.L., Griffin N.W., Lombard V., Henrissat B., Bain J.R. (2013). Gut microbiota from twins discordant for obesity modulate metabolism in mice. Science.

[2] Frank D.N., St Amand A.L., Feldman R.A., Boedeker E.C., Harpaz N., Pace N.R. (2007). Molecular-phylogenetic characterization of microbial community imbalances in human inflammatory bowel diseases. Proc. Natl. Acad. Sci. USA.

[3] Chevalier C., Stojanovic O., Colin D.J., Suarez-Zamorano N., Tarallo V., Veyrat-Durebex C., Rigo D., Fabbiano S., Stevanovic A., Hagemann S. (2015). Gut Microbiota Orchestrates Energy Homeostasis during Cold. Cell.

[4] Worthmann A., John C., Ruhlemann M.C., Baguhl M., Heinsen F.A., Schaltenberg N., Heine M., Schlein C., Evangelakos I., Mineo C. (2017). Cold-induced conversion of cholesterol to bile acids in mice shapes the gut microbiome and promotes adaptive thermogenesis. Nat. Med..

[5] Xu Z., Huang C., Turner L.R., Su H., Qiao Z., Tong S. (2013). Is diurnal temperature range a risk factor for childhood diarrhea?. PLoS ONE.

[6] Thiam S., Diene A.N., Sy I., Winkler M.S., Schindler C., Ndione J.A., Faye O., Vounatsou P., Utzinger J., Cisse G. (2017). Association between Childhood Diarrhoeal Incidence and Climatic Factors in Urban and Rural Settings in the Health District of Mbour, Senegal. Int. J. Environ. Res. Public Health.

[7] Kelley K.W. (1980). Stress and immune function: A bibliographic review. Ann. Rech. Vet..

[8] Laine T.M., Lyytikainen T., Yliaho M., Anttila M. (2008). Risk factors for post-weaning diarrhoea on piglet producing farms in Finland. Acta Vet. Scand..

[9] LaVoy E.C., McFarlin B.K., Simpson R.J. (2011). Immune responses to exercising in a cold environment. Wilderness Environ. Med..

[10] Yang X.J., Li W.L., Feng Y., Yao J.H. (2011). Effects of immune stress on growth performance, immunity, and cecal microflora in chickens. Poult. Sci..

[11] Kaushik S., Kaur J. (2005). Effect of chronic cold stress on intestinal epithelial cell proliferation and inflammation in rats. Stress.

[12] Rogier E.W., Frantz A.L., Bruno M.E., Wedlund L., Cohen D.A., Stromberg A.J., Kaetzel C.S. (2014). Lessons from mother: Long-term impact of antibodies in breast milk on the gut microbiota and intestinal immune system of breastfed offspring. Gut Microbes.

[13] Bauerl C., Collado M.C., Zuniga M., Blas E., Perez Martinez G. (2014). Changes in cecal microbiota and mucosal gene expression revealed new aspects of epizootic rabbit enteropathy. PLoS ONE.

[14] Kogut M.H., Arsenault R.J. (2016). Editorial: Gut health: The new paradigm in food animal production. Front. Vet. Sci..

[15] Heaney D.P., Jnb S. (1986). Effects of warm versus cold milk replacers and of free-choice hay postweaning on performance of artificially reared lambs. Can. J. Anim. Sci.

[16] Osborne V.R., Hacker R.R., Mcbride B.W. (2002). Effects of heated drinking water on the production responses of lactating Holstein and Jersey cows. Can. J. Anim. Sci..

[17] Maynard C.L., Elson C.O., Hatton R.D., Weaver C.T. (2012). Reciprocal interactions of the intestinal microbiota and immune system. Nature.

[18] Young V.B. (2012). The intestinal microbiota in health and disease. Curr. Opin. Gastroenterol..

[19] Combes S., Michelland R.J., Monteils V., Cauquil L., Soulie V., Tran N.U., Gidenne T., Fortun-Lamothe L. (2011). Postnatal development of the rabbit caecal microbiota composition and activity. FEMS Microbiol. Ecol..

[20] Kundu P., Blacher E., Elinav E., Pettersson S. (2017). Our gut microbiome: The evolving inner self. Cell.

[21] Tremaroli V., Backhed F. (2012). Functional interactions between the gut microbiota and host metabolism. Nature.

[22] Jobin C. (2014). GPR109a: The missing link between microbiome and good health?. Immunity.

[23] Singh N., Gurav A., Sivaprakasam S., Brady E., Padia R., Shi H., Thangaraju M., Prasad P.D., Manicassamy S., Munn D.H. (2014). Activation of Gpr109a, receptor for niacin and the commensal metabolite butyrate, suppresses colonic inflammation and carcinogenesis. Immunity.

[24] Hamer H.M., Jonkers D., Venema K., Vanhoutvin S., Troost F.J., Brummer R.J. (2008). Review article: The role of butyrate on colonic function. Aliment. Pharmacol. Ther..

[25] Huang C., Song P., Fan P., Hou C., Thacker P., Ma X. (2015). Dietary sodium butyrate decreases postweaning diarrhea by modulating intestinal permeability and changing the bacterial communities in weaned piglets. J. Nutr..

[26] Ma X., Fan P.X., Li L.S., Qiao S.Y., Zhang G.L., Li D.F. (2012). Butyrate promotes the recovering of intestinal wound healing through its positive effect on the tight junctions. J. Anim. Sci..

[27] Zhang X.Y., Sukhchuluun G., Bo T.B., Chi Q.S., Yang J.J., Chen B., Zhang L., Wang D.H. (2018). Huddling remodels gut microbiota to reduce energy requirements in a small mammal species during cold exposure. Microbiome.

[28] Agazzi A. (2015). The beneficial role of probiotics in monogastric animal nutrition and health. J. Dairy Vet. Anim. Res..

[29] Huff G.R., Huff W.E., Rath N.C., El-Gohary F.A., Zhou Z.Y., Shini S. (2015). Efficacy of a novel prebiotic and a commercial probiotic in reducing mortality and production losses due to cold stress and Escherichia coli challenge of broiler chicks 1. Poult. Sci..

[30] Schnupf P., Sansonetti P.J. (2012). Quantitative RT-PCR profiling of the rabbit immune response: Assessment of acute Shigella flexneri infection. PLoS ONE.

[31] Whiting R.C., Jenkins R.K. (1981). Comparison of rabbit, beef, and chicken meats for functional-properties and frankfurter processing. J. Sci. Food Agric..

[32] Zhu Y., Wang C., Li F. (2015). Impact of dietary fiber/starch ratio in shaping caecal microbiota in rabbits. Can. J. Microbiol..

[33] Larzul C., de Rochambeau H. (2005). Selection for residual feed consumption in the rabbit. Livest. Prod. Sci..

[34] Zanuzzi C.N., Fontana P.A., Barbeito C.G., Portiansky E.L., Gimeno E.J. (2008). Paneth cells: Histochemical and morphometric study in control and Solanum glaucophyllum intoxicated rabbits. Eur. J. Histochem..

[35] Wang W., Yang Q., Sun Z., Chen X., Yang C., Ma X. (2015). Editorial: Advance of interactions between exogenous natural bioactive peptides and intestinal barrier and immune responses. Curr. Protein Pept. Sci..

[36] Broderick G.A., Kang J.H. (1980). Automated simultaneous determination of ammonia and total amino acids in ruminal fluid and in vitro media. J. Dairy Sci..

[37] Edgar R.C., Haas B.J., Clemente J.C., Quince C., Knight R. (2011). UCHIME improves sensitivity and speed of chimera detection. Bioinformatics.

[38] Edgar R.C. (2010). Search and clustering orders of magnitude faster than BLAST. Bioinformatics.

[39] Bokulich N.A., Subramanian S., Faith J.J., Gevers D., Gordon J.I., Knight R., Mills D.A., Caporaso J.G. (2013). Quality-filtering vastly improves diversity estimates from Illumina amplicon sequencing. Nat. Methods.

[40] Caporaso J.G., Kuczynski J., Stombaugh J., Bittinger K., Bushman F.D., Costello E.K., Fierer N., Pena A.G., Goodrich J.K., Gordon J.I. (2010). QIIME allows analysis of high-throughput community sequencing data. Nat. Methods.

[41] Arumugam M., Raes J., Pelletier E., Le Paslier D., Yamada T., Mende D.R., Fernandes G.R., Tap J., Bruls T., Batto J.M. (2011). Enterotypes of the human gut microbiome. Nature.

[42] Schloss P.D., Westcott S.L., Ryabin T., Hall J.R., Hartmann M., Hollister E.B., Lesniewski R.A., Oakley B.B., Parks D.H., Robinson C.J. (2009). Introducing mothur: Open-source, platform-independent, community-supported software for describing and comparing microbial communities. Appl. Environ. Microbiol..

[43] Shannon P., Markiel A., Ozier O., Baliga N.S., Wang J.T., Ramage D., Amin N., Schwikowski B., Ideker T. (2003). Cytoscape: A software environment for integrated models of biomolecular interaction networks. Genome Res..

[44] Campbell J.M., Crenshaw J.D., Polo J. (2013). The biological stress of early weaned piglets. J. Anim. Sci. Biotechnol..

[45] Francino M.P. (2014). Early development of the gut microbiota and immune health. Pathogens.

[46] Shen X., Miao J., Wan Q., Wang S., Li M., Pu F., Wang G., Qian W., Yu Q., Marotta F. (2018). Possible correlation between gut microbiota and immunity among healthy middle-aged and elderly people in southwest China. Gut Pathog..

[47] Shaw K.A., Bertha M., Hofmekler T., Chopra P., Vatanen T., Srivatsa A., Prince J., Kumar A., Sauer C., Zwick M.E. (2016). Dysbiosis, inflammation, and response to treatment: A longitudinal study of pediatric subjects with newly diagnosed inflammatory bowel disease. Genome Med..

[48] Ling Z., Liu F., Shao L., Cheng Y., Li L. (2017). Dysbiosis of the Urinary Microbiota Associated With Urine Levels of Proinflammatory Chemokine Interleukin-8 in Female Type 2 Diabetic Patients. Front. Immunol..

[49] Qiu Z., Yang H., Rong L., Ding W., Chen J., Zhong L. (2017). Targeted Metagenome Based Analyses Show Gut Microbial Diversity of Inflammatory Bowel Disease patients. Indian J. Microbiol..

[50] Luna R.A., Oezguen N., Balderas M., Venkatachalam A., Runge J.K., Versalovic J., Veenstra-VanderWeele J., Anderson G.M., Savidge T., Williams K.C. (2017). Distinct microbiome-neuroimmune signatures correlate with functional abdominal pain in children with autism spectrum disorder. Cell. Mol. Gastroenterol. Hepatol..

[51] Wu A., Duan J., Liu S., Meng X., Zhou P., Dou Q., Li C. (2017). Gut microbiota composition related with clostridium difficile-positive diarrhea and C. difficile type (A+B+, A-B+, And A-B-) in ICU hospitalized patients. BioRxiv.

[52] De Cruz P., Kang S., Wagner J., Buckley M., Sim W.H., Prideaux L., Lockett T., McSweeney C., Morrison M., Kirkwood C.D. (2015). Association between specific mucosa-associated microbiota in Crohn’s disease at the time of resection and subsequent disease recurrence: A pilot study. J. Gastroenterol. Hepatol..

[53] Chang L., Neu J. (2015). Early factors leading to later obesity: Interactions of the microbiome, epigenome, and nutrition. Curr. Probl. Pediatr. Adolesc. Health Care.

[54] Wang Y., Ames N.P., Tun H.M., Tosh S.M., Jones P.J., Khafipour E. (2016). High molecular weight barley beta-glucan alters gut microbiota toward reduced cardiovascular disease risk. Front. Microbiol..

[55] Chen L., Xu Y., Chen X., Fang C., Zhao L., Chen F. (2017). The Maturing development of gut microbiota in commercial piglets during the weaning transition. Front. Microbiol..

[56] Le Chatelier E., Nielsen T., Qin J., Prifti E., Hildebrand F., Falony G., Almeida M., Arumugam M., Batto J.M., Kennedy S. (2013). Richness of human gut microbiome correlates with metabolic markers. Nature.

[57] Konopka A. (2009). What is microbial community ecology?. ISME J..

[58] Reichardt N., Duncan S.H., Young P., Belenguer A., McWilliam Leitch C., Scott K.P., Flint H.J., Louis P. (2014). Phylogenetic distribution of three pathways for propionate production within the human gut microbiota. ISME J..

[59] Fung K.Y., Cosgrove L., Lockett T., Head R., Topping D.L. (2012). A review of the potential mechanisms for the lowering of colorectal oncogenesis by butyrate. Br. J. Nutr..

[60] Wilson A.J., Chueh A.C., Togel L., Corner G.A., Ahmed N., Goel S., Byun D.S., Nasser S., Houston M.A., Jhawer M. (2010). Apoptotic sensitivity of colon cancer cells to histone deacetylase inhibitors is mediated by an Sp1/Sp3-activated transcriptional program involving immediate-early gene induction. Cancer Res..

[61] Blas E., Gidenne T., De Blas C., Wiseman J. (2010). Digestion of starch and sugars. The Nutrition of the Rabbit.

[62] Degen A.A., Young B.A. (1984). Effects of ingestion of warm, cold and frozen water on heat-balance in cattle. Can. J. Anim. Sci.

[63] Nicol A.M., Young B.A. (1990). Short-term thermal and metabolic responses of sheep to ruminal cooling: Effects of level of cooling and physiological state. Can. J. Anim. Sci..

[64] Louis P., Duncan S.H., McCrae S.I., Millar J., Jackson M.S., Flint H.J. (2004). Restricted distribution of the butyrate kinase pathway among butyrate-producing bacteria from the human colon. J. Bacteriol..

[65] Bedford A., Gong J. (2018). Implications of butyrate and its derivatives for gut health and animal production. Anim. Nutr..

[66] Sleeth M.L., Thompson E.L., Ford H.E., Zac-Varghese S.E., Frost G. (2010). Free fatty acid receptor 2 and nutrient sensing: A proposed role for fibre, fermentable carbohydrates and short-chain fatty acids in appetite regulation. Nutr. Res. Rev..

[67] Flynn N.E., Wu G. (1997). Glucocorticoids play an important role in mediating the enhanced metabolism of arginine and glutamine in enterocytes of postweaning pigs. J. Nutr..

[68] Silverman M.N., Sternberg E.M. (2012). Glucocorticoid regulation of inflammation and its functional correlates: From HPA axis to glucocorticoid receptor dysfunction. Ann. N. Y. Acad. Sci..

[69] Lacey J.M., Wilmore D.W. (2010). Is glutamine a conditionally essential amino acid?. Nutr. Rev..

[70] Zhang X. (2015). Molecular sensors and modulators of thermoreception. Channels.

[71] Galindo-Villegas J., Garcia-Moreno D., de Oliveira S., Meseguer J., Mulero V. (2012). Regulation of immunity and disease resistance by commensal microbes and chromatin modifications during zebrafish development. Proc. Natl. Acad. Sci. USA.

