# Supplementary material for: Drinking Warm Water Improves Growth Performance and Optimizes the Gut Microbiota in Early Postweaning Rabbits during Winter
Source: Animals (Basel). 2019 Jun 12;9(6):346. doi: 10.3390/ani9060346 (PMC6616395; doi:10.3390/ani9060346)
Supplement: Supplementary file 1 [file animals-09-00346-s001.pdf]

Supplementary Material

Supplementary Figures

Figure S1. Taxonomic alteration of cecum microbiota in rabbits from different groups at the phyla and genus level. CW, cold water group; WW, warm water group.

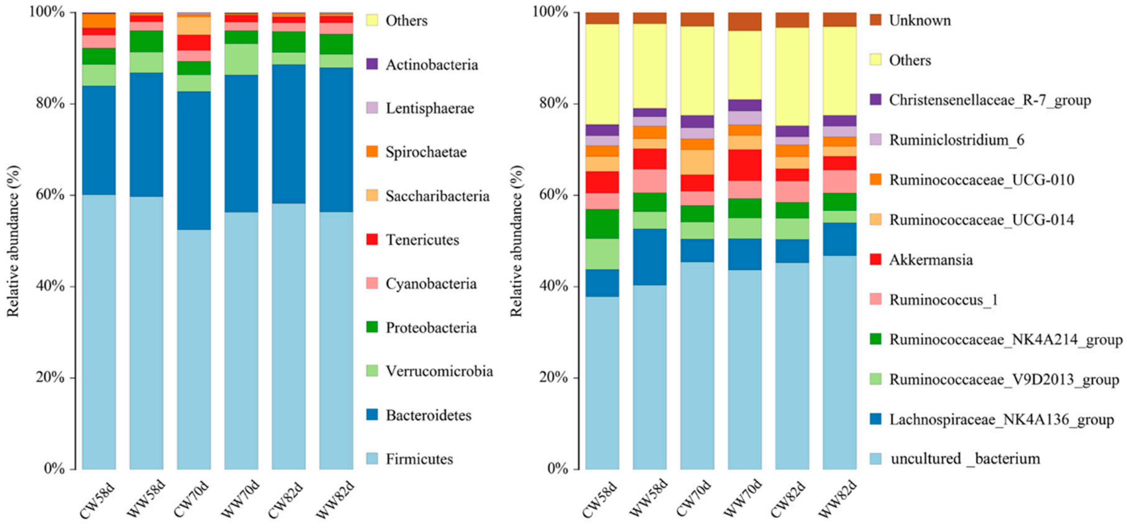

Figure S2. (**A**) Genus differentially represented between the early postweaning (day 58) and subadult rabbits (day 82) identified by LEfSe (linear discriminant analysis score >2, P < 0.05). (**B**) Significantly different genera among the early postweaning (day 58) and subadult rabbits (day 82) are shown, with P < 0.05 by Wilcoxon rank-sum test. CW, cold water group; WW, warm water group.

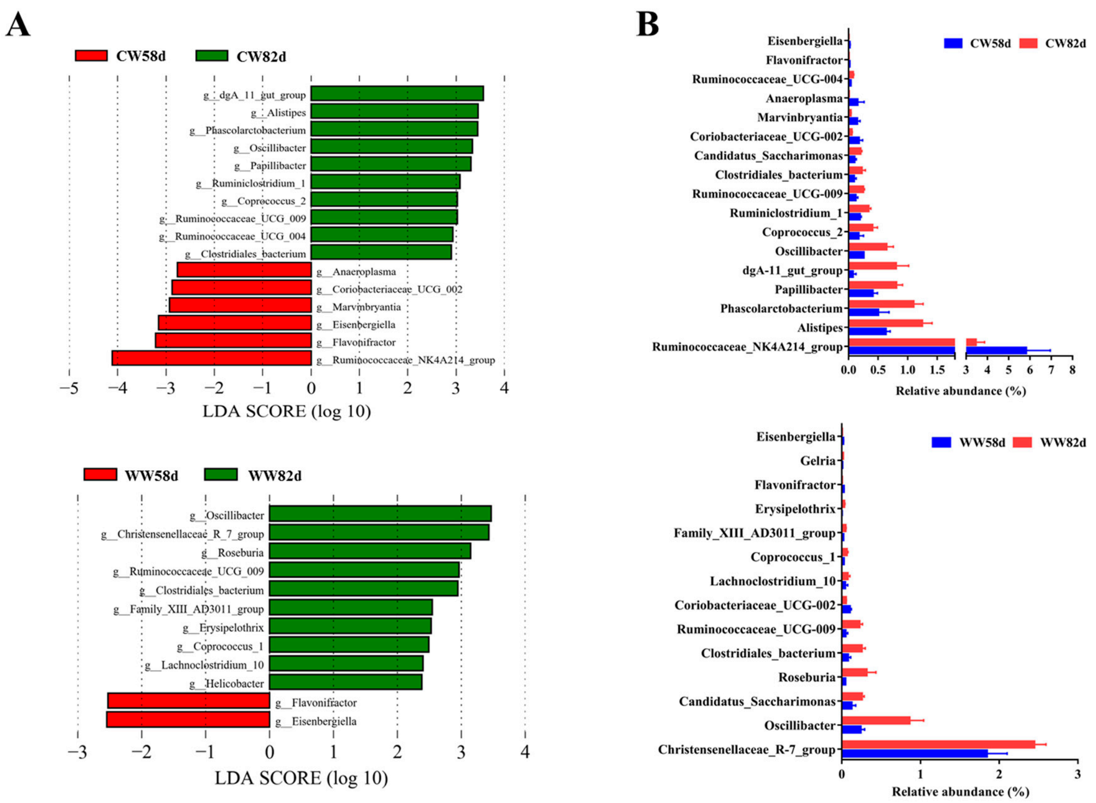

Supplementary Tables

Table S1. Composition of the basal diets

Item	Content	Item	Content
Ingredients		Chemical composition	
Alfalfa meal	31.80	Dry matter (g/kg feed)	868.00
Corn	27.60	Crude protein (g/kg DM)	202.00
Soybean meal	17.70	Neutral detergent fibre (g/kg	318.00
Wheat bran	20.00	Ether extract (g/kg DM)	30.00
Premix[a]	0.15	Digestible energy (MJ/kg	11.89
Salt	0.40	Ca (g/kg DM)	10.40
Limestone	0.39	Total phosphorus (g/kg DM)	7.40
dl-Methionine	0.41	Lysine (g/kg DM)	9.80
Lysine	0.05	Methionine + cysteine (g/kg	9.30
Calcium hydro phosphate	1.30	Threonine (g/kg DM)	9.20
Threonine	0.20		
Total	100.00		

[a] Premix provided per kg of diet: 12 000 IU of vitamin A; 2500 IU of vitamin D3; 40 mg of vitamin E; 2.0 mg of vitamin K; 2.0 mg of vitamin B1; 4 mg of vitamin B2; 2.0 mg of vitaminB6; 0.01 mg of vitamin B12; 0.06 mg of biotin; 50 mg of niacin; 0.3 mg of folic acid; 10 mg of d-pantothenic acid; 1000 mg of choline; 40 mg of Zn; 10 mg of Cu; 30 mg of Mn; 50 mg of Fe; 0.5 mg of I; 0.2 mg of Se; 0.5 mg of Co.

Table S2. Primers used for PCR

Gene	Forward primer (5' to 3')	reverse primer (5' to 3')
TGF-β1	CACAGCATGAACCGACCCTTC	AGTTGGCGTGGTAGCCCTTG
IL-1β	GCCGATGGTCCCAATTACAT	TCCAGAGCCACAACGACTGA
IL-10	AAGCCTTGTCGGAGATGAT	GCTTTGTAGACGCCTTCCTC
IL-12	ACCTCCTCTATGGTGACCC	CAGCTCCACTGTTGGAATTC
pIgR2	ACTGGTGCAAGTGGAATGACC	CGCAACCTCTTCAAACTCGTG
GRα	GGGAAGGAAACTCCAGTCAGAAC	GATTGGTGATGATTTCAGCTAGCA
occludin	AGAGTCCTACAAGTCCACACCG	TCGTAGTGGTCTTGCTCTGATCTC
claudin-1	ATAGCAATCTTTGTGGCCACTGTT	CCGCATCTTTTGCTCCTCATC
GAPDH	TGGTGAAGGTCGGAGTGAAC	ATGTAGTGGAGGTCAATGAATGG
